# Efficient Metal‐Oriented Electrodeposition of a Co‐Based Metal‐Organic Framework with Superior Capacitive Performance

**DOI:** 10.1002/cssc.202200644

**Published:** 2022-05-24

**Authors:** Yan Han, Jian Cui, Yue Yu, Yunfeng Chao, Dejun Li, Caiyun Wang, Gordon G. Wallace

**Affiliations:** ^1^ Energy & Materials Engineering Centre College of Physics and Materials Science Tianjin Normal University Tianjin 300387 P. R. China; ^2^ ARC Centre of Excellence for Electromaterials Science Intelligent Polymer Research Institute University of Wollongong New South Wales 2500 Australia; ^3^ Henan Institute of Advanced Technology Zhengzhou University Zhengzhou 450052 P. R. China

**Keywords:** carbon fiber, cobalt, electrodeposition, metal-organic frameworks, supercapacitors

## Abstract

An efficient cathodic electrodeposition method is developed for coating Co‐based metal‐organic frameworks (Co‐MOF) on carbon fiber cloth (CFC), a widely used substrate in energy fields. The use of a highly active Co metal surface enables nucleation and growth of Co‐MOF in 3D rodlike crystal bundles. When used as a binder‐free electrode (Co‐MOF/CFC) for supercapacitors, it shows a high areal capacitance of 1784 mF cm^−2^ at 1 mA cm^−2^, good cycling stability and excellent rate capability. The assembled asymmetric all‐solid‐state supercapacitor device (Co‐MOF/CFC//AC) delivers a high energy density and power density. This work may open up an effective approach to realize the electrosynthesis of MOF films, promoting use in energy storage and conversion fields.

## Graphical abstract

**Co‐MOF it**: An efficient metal‐oriented cathodic electrodeposition method is developed for coating Co‐MOF on carbon fiber cloth (CFC), which is realized by using a highly active Co metal surface modifier that enables nucleation and growth of Co‐MOF in 3D rodlike crystal bundles. As a binder‐free electrode for supercapacitors, Co‐MOF/CFC shows high areal capacitance, good cycling stability, and excellent rate capability.

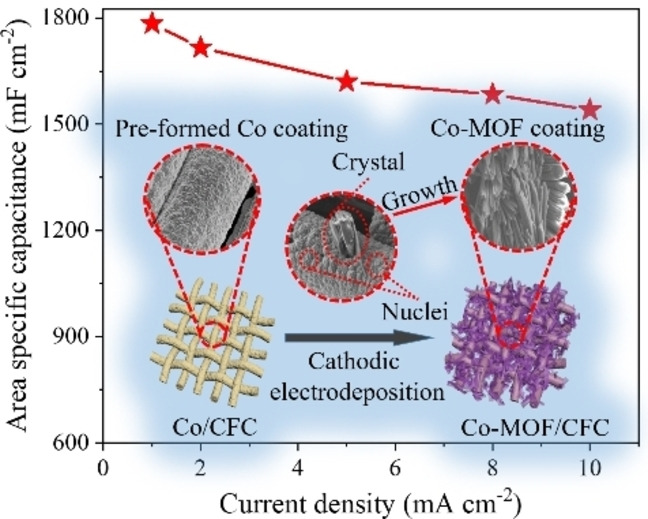

## Introduction

Metal‐organic frameworks (MOFs) are promising supercapacitor electrode materials due to their large surface area, well‐defined pore structure and the redox‐active metal centers.[^1^, ^2^, ^3^, ^4^, ^5^] However, the application of MOFs in supercapacitors is largely hindered by their poor electrical conductivity and low exposure of active metal centers. To resolve these problems, directly growing MOFs onto conductive substrates is an effective strategy that imparts fast electron transport as well as avoiding MOF aggregation ensuring high electrochemically active surface area.[^6^, ^7^, ^8^, ^9^] Removing the need for binders and conductive additives decreases the “dead weight” and increases utility.

Electrochemical deposition is an elegant way to grow MOF coatings directly on conductive substrates, which can be performed at room temperature in a short timeframe and an easily controllable manner without the use of expensive specialized equipment.[^10^, ^11^] MOF coatings can be constructed through both oxidative and reductive reactions. For example, on metal anodes (e. g., Cu, Co, Zn and Ni), MOF coatings were deposited under high potentials through the binding of oxidized metal ions and organic ligands (e. g., HKUST‐1, ZIF‐67, ZIF‐8, and 2D−Co−Ni mixed MOF).[^12^, ^13^, ^14^, ^15^] During this process, the anodes were corroded to provide metal ions for the formation of MOFs, thus this anodic method places a limitation on the choice of electrode substrates. In the cathodic method both metal cations and organic ligands are present in the electrolyte solution.[^16^, ^17^, ^18^] Under cathodic potentials, probases, such as NO_3_^−^, Et_3_NH^+^ or H_2_O, are reduced near the electrode surface and the local pH values increase, which cause the deprotonation of organic ligands and the subsequent reaction with metal cations to form MOF coatings on electrode surfaces. In theory, MOF coatings could be formed on any conductive substrates through this method. However, owing to the general incompatibility between the substrates and MOF species, one main challenge is how to achieve robust growth of well‐intergrown MOF coatings on conductive substrates.

The decoration of organic or inorganic modifiers (e. g., polydopamine, 3‐aminopropyltriethoxysilane, ZnO, Cu_2_O and ZnAl−CO_3_) on the substrate surface is commonly used to improve the heterogeneous nucleation and growth of MOF membranes.[^19^, ^20^, ^21^, ^22^, ^23^, ^24^] Compared with organic modifiers, inorganic modifiers have higher thermal stability, high affinity with the substrate, and are environment‐ and eco‐friendly. However, the reported methods for introduction of inorganic modifiers are complicated and time‐consuming, and typically involve calcination or hydrothermal treatment.[^21^, ^22^, ^23^] A simple and facile method is highly desired. Moreover, electrodeposition of MOFs on carbon‐based materials (e. g., carbon cloth, graphite paper), which are widely used as substrates in energy devices, has not yet been reported.

Herein, we report an innovative facile and efficient cathodic electrodeposition of cobalt‐based metal‐organic framework (Co‐MOF) coating on flexible carbon fiber cloth (CFC). This is realized by using a preformed highly reactive metal‐oriented surface modifier that provides nucleation sites for continuous growth of MOF. This method can be easily extended to cathodic electrodeposition of different MOFs on the extensively used substrates in industry (e. g., carbon paper, nickel foam and stainless steel). The obtained Co‐MOF coating is composed of 3D rodlike crystal bundles tightly bonded onto CFC. These Co‐MOF/CFC electrodes are directly used as binder‐free and integrated electrodes for supercapacitors and have displayed excellent performance.

## Results and Discussion

### Characterization of Co‐MOF/CFC complex

**Figure 1:**
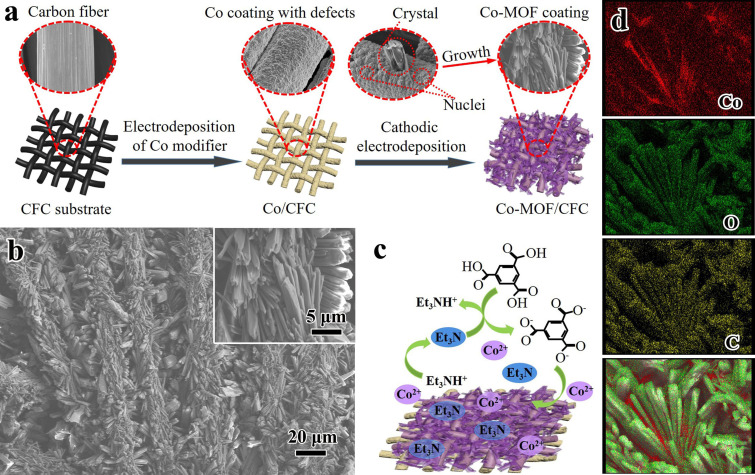
Electrodeposition of Co‐MOF on carbon fiber cloth (Co‐MOF/CFC): (a) Schematic representation of the electrodeposition procedure. (b) SEM images of Co‐MOF/CFC deposited for 15 min. (c) Proposed mechanism for the cathodic electrodeposition. (d) EDS mapping images for Co, O, and C.

The synthetic scheme for continuous Co‐MOF coating on CFC is depicted in Figure 1a. The pre‐electrodeposited cobalt coating played a crucial role in the subsequent cathodic electrodeposition of the MOFs. We had tried to synthesize Co‐MOF coating on bare CFC under different conditions, but all failed (see the Supporting Information, Figure S1). It may be attributed to the difficulty in heterogeneous nucleation and growth of Co‐MOF crystals on bare CFC.^[24]^ The cobalt coating acted as the structure/interface coupling bridge providing active sites for the nucleation and growth of MOF crystals, which ensured the stable immobilization of MOF coating on carbon fibers. The XRD pattern of Co‐modified CFC (Co/CFC) confirmed the formation of pure Co phase (Figure S2; JCPDF no. 1‐1254). Compared with bare CFC that had striations along the carbon fiber axis, Co/CFC featured with an uneven and rough surface with abundant defects (Figure S3). The fresh Co/CFC was then used for the growth of Co‐MOF, which was conducted at the deposition potential of −2.5 V for different durations. After 1 min, protuberant particles covered the surface of the carbon fibers and some rodlike crystals were formed (Figure S4a, b). With increased deposition time, more rodlike crystals with different sizes were produced and were randomly distributed on carbon fibers (Figure S4c–f). When the deposition time reached 15 min, a uniform Co‐MOF coating was obtained (Figure 1b). It was composed of 3D rodlike crystals in length of 5–20 μm and thickness of 1–5 μm, which formed bundles and were packed closely covering the whole carbon fiber surface of CFC. This method achieved a high mass loading (up to 15 mg cm^−2^). The relatively low number of large crystals led to the assumption that the growth process was more pronounced than the nucleation during the electrochemical deposition procedure.^[14]^ With increased deposition time, the coating thickness increased and some crystals were grown into the interstitial volume of the fibers. These materials easily separated from the fiber/CFC and dropped into the solution because of the poor adhesion (Figure S4g, h).

The proposed mechanism of cathodic electrodeposition of Co‐MOF coating on the Co‐modified CFC is presented in Figure 1c. The electrodeposited Co coating had considerable reactivity ascribed to the intrinsic defects arising from the electrodeposition at low temperature (e. g., room temperature).^[25]^ This Co‐modified CFC was used directly for the in situ growth of MOF coating without a pre‐activation treatment, the commonly used for the anodic electrodeposition of MOF on Co substrate.[^8^, ^15^] With the application of a constant potential, the reduction of triethylammonnium (Et_3_NH^+^) to trimethylamine (Et_3_N) and H_2_ increased the local pH values near the cathode surface, which caused the deprotonation of neutral ligands (H_3_BTC). This triggered the self‐assembly of Co^2+^ at the negative potential and deprotonated ligand (BTC^3−^) to form Co‐MOF crystal nucleus anchoring on the substrate of Co/CFC, which tended to start from defects of the Co coating. Consequently, nucleation was initiated next to the first nucleus and in the meantime those already nucleated crystals kept growing to several microns, eventually forming a dense, intergrown and continuous layer. The elemental mapping by energy‐dispersive X‐ray spectroscopy (EDS; Figure 1d) clearly verified the coexistence and relatively homogeneous distribution of Co, O and C elements on Co‐MOF/CFC. In addition, the much higher percentage of cobalt signature between Co‐MOF crystals indicated that the pre‐deposited cobalt coating was retained along with Co‐MOF formation.

**Figure 2:**
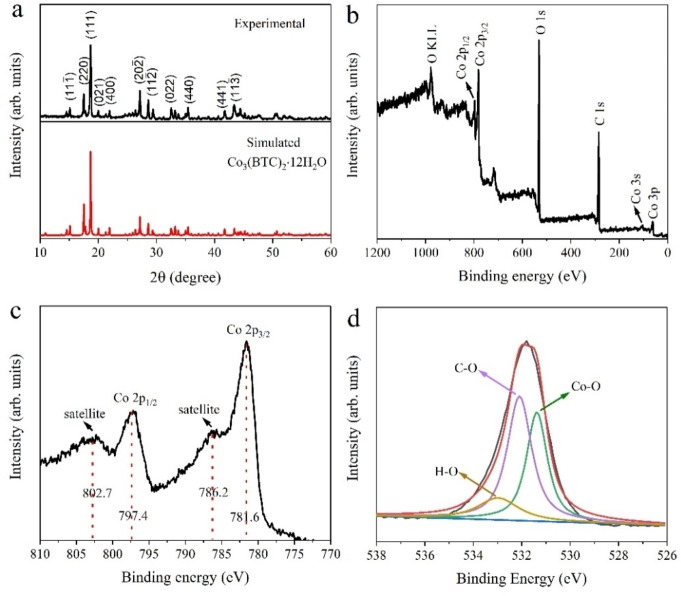
(a) XRD patterns of Co‐MOF/CFC and simulated Co_3_(BTC)_2_ ⋅ 12H_2_O. (b–d) XPS spectra of full scan survey (b), Co2p (c), and O1s (d) for Co‐MOF/CFC.

The as‐synthesized Co‐MOF/CFC deposited for an optimal time of 15 min was further characterized by XRD, XPS and FTIR. The XRD pattern in Figure 2a showed that all diffraction peaks corresponded well to the standard pattern of monoclinic Co_3_(BTC)_2_ ⋅ 12H_2_O (space groups C2) and agreed with the simulated pattern based on the single crystal structure data (CCDC‐1274034), confirming the high purity of Co‐MOF coating.[^26^, ^27^] The high reflection intensities and sharp peaks confirmed a well‐crystallized MOF structure. The XPS wide survey spectrum of Co‐MOF/CFC (Figure 2b) indicated the existence of Co, C and O elements, which are consistent with the EDS analysis. The high‐resolution Co2p XPS spectrum (Figure 2c) showed the characteristic Co2p_1/2_ peak and its satellite peak at 797.4 and 802.7 eV, while the characteristic Co2p_3/2_ peak and its satellite peak located at 781.6 and 786.2 eV, respectively. The energy difference between Co2p_3/2_ and Co2p_1/2_ peaks was approximately 15.8 eV, which proves that Co ions were predominantly in the Co^2+^ state in Co‐MOF/CFC.[^28^, ^29^] The high‐resolution O1s spectrum (Figure 2d) can be divided into three peaks at 531.4, 532.1 and 533.0 eV. The species at 531.4 and 532.1 eV are attributed to the oxygen atoms on Co−O bands and O=C−O groups of terephthalic acid linkers, whereas the species at 533.0 eV is assigned to coordinated water. The high‐resolution C1s spectrum is also shown in Figure S5. Two characteristic peaks at 288.5 and 284.8 eV were respectively related to O=C−O and C−C/C=C bond, further evidencing the presence of terephthalic acid linkers.^[30]^

**Figure 3:**
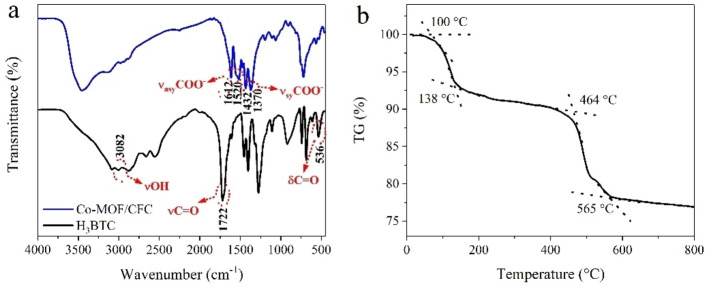
(a) FTIR spectra of Co‐MOF/CFC and H_3_BTC. (b) TG curve of Co‐MOF/CFC under air.

The FTIR spectra of Co‐MOF/CFC and H_3_BTC (Figure 3a) presented two important changes between them. First, the characteristic bands of nonionized carboxyl groups (*δ*_C=O_=536 cm^−1^; $\bar{\nu }$_C=O_=1722 cm^−1^; $\bar{\nu }$_OH_=3082 cm^−1^) for H_3_BTC disappeared in Co‐MOF/CFC, revealing the complete deprotonation of H_3_BTC linker upon reaction with Co^2+^ forming MOF. Second, new strong bands arose in the regions of 1612–1520 cm^−1^ and 1432–1370 cm^−1^, which can be assigned to the asymmetric and symmetric stretching vibrations of carboxylate anions. This provided additional evidence of the coordination of organic ligand with Co^2+^ ions. Furthermore, the broadband at around 3500 cm^−1^ corresponding to the stretching vibration of O−H bond indicates the presence of coordinated water molecules in the sample. On the basis of these above results, cobalt ions had been coordinated with the H_3_BTC ligand to form the Co‐MOF frame structure.

The thermal stability of Co‐MOF/CFC was investigated by TGA under air. As shown in Figure 3b, two weight‐loss steps were observed. The first weight loss took place between 100 and 140 °C, which corresponded to the removal of coordinated H_2_O molecules and adsorbed solvent. The weight was kept steady up to 460 °C. The second and main weight loss from 460 to 570 °C may be ascribed to the degradation of tricarboxylate linkers releasing CO and CO_2_, followed by the complete decomposition of Co‐MOF framework to cobalt oxides.[^29^, ^31^] These results indicate that the framework of Co‐MOF was stable up to 460 °C.

### Electrochemical characteristics

**Figure 4:**
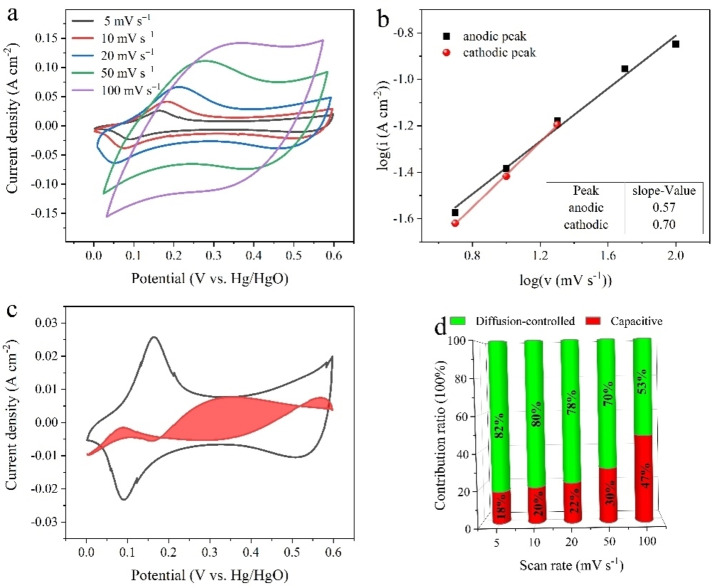
(a) CV curves of the Co‐MOF/CFC electrode at the scan rates ranging from 5 to 100 mV s^−1^. (b) Determination of *b* value using the relationship between peak current and scan rate. (c) Capacitive contribution (red area) at 5 mV s^−1^. (d) Contribution ratio of surface‐limited process and diffusion‐limited process at different scan rate

The electrochemical properties of Co‐MOF/CFC supercapacitor electrodes were evaluated by using a three‐electrode configuration in 1 m LiOH aqueous electrolyte. The CV curves of Co‐MOF/CFC and Co/CFC electrodes at 20 mV s^−1^ (Figure S6) showed that Co‐MOF/CFC displayed much larger CV current response than Co/CFC, which verified the major contribution from Co‐MOF. The representative CV curves of Co‐MOF/CFC at different scan rates (Figure 4a) showed a couple of redox peaks during the anodic and cathodic sweeps, indicating that the electrochemical capacitance of the electrode mainly came from faradaic pseudocapacitance ascribed to the valence state change of cobalt occurred in two quasi‐reversible processes [Eqs. (1) and (2)], similar to some reported MOF‐based electrode materials.[^32^, ^33^, ^34^, ^35^]

(1) $$\mathrm{Co}{}^{\mathrm{II}}{}_{s}+\mathrm{OH}{}^{-}↔\mathrm{Co}{}^{\mathrm{II}}\left(\mathrm{OH}\right){}_{\mathrm{ad}}+e{}^{-}$$

(2) $$\mathrm{Co}{}^{\mathrm{II}}\left(\mathrm{OH}\right){}_{\mathrm{ad}}↔\mathrm{Co}{}^{\mathrm{III}}\left(\mathrm{OH}\right){}_{\mathrm{ad}}+e{}^{-}$$

With increasing scan rate from 5 to 100 mV s^−1^, the cathodic peaks shifted towards negative potential and anodic peaks shifted towards positive potential, which may be explained by the limited charge transfer kinetics.

The electrochemical reaction kinetics can be analyzed by using Equation 3:[^36^, ^37^]

(3) $$i=av{}^{b}$$

where *a* and *b* are adjustable parameters, *i* and *v* are peak current and scan rate, respectively. The value of *b* gives an insight on the charge storage mechanism, which can be obtained from the slope of log(*i*) vs. log(*v*) curve. In general, the *b* value close to 1 indicates the main contribution is from surface‐limited capacitive process, whereas the *b* value close to 0.5 means the main contribution is from diffusion‐controlled intercalation process. The corresponding *b* values of Co‐MOF/CFC electrode for anodic and cathodic peaks were calculated to be 0.57 and 0.70, respectively (Figure 4b). Both *b* values approached 0.5, proving the electrochemical process of Co‐MOF/CFC was governed by diffusion‐controlled intercalation behavior. In addition, the percentage of capacitive contribution and diffusion‐controlled contribution at different scan rates were further analyzed by using Equation 4:[^38^, ^39^, ^40^]

(4) $$i\left(V\right)=k{}_{1}v+k{}_{2}v{}^{1/2}$$

where *k*_1_ and *k*_2_ are scan rate independent constants, *k*_1_ *v* and *k*_2_ *v* ^1/2^ correspond to the current contributions from surface‐limited capacitive behavior and diffusion‐controlled intercalation behavior. As shown in Figure 4c, the contribution of capacitive behavior (red shaded region) of the Co‐MOF/CFC electrode was around 18 % at 5 mV s^−1^. As the scan rate increased from 5 to 100 mV s^−1^, the capacitive effects gradually increased from 18 % to 47 %, meanwhile the diffusion‐controlled intercalation contributions decreased, which was ascribed to the shorter time for electrolyte ions to diffuse into the material at high scan rates (Figure 4d).

**Figure 5:**
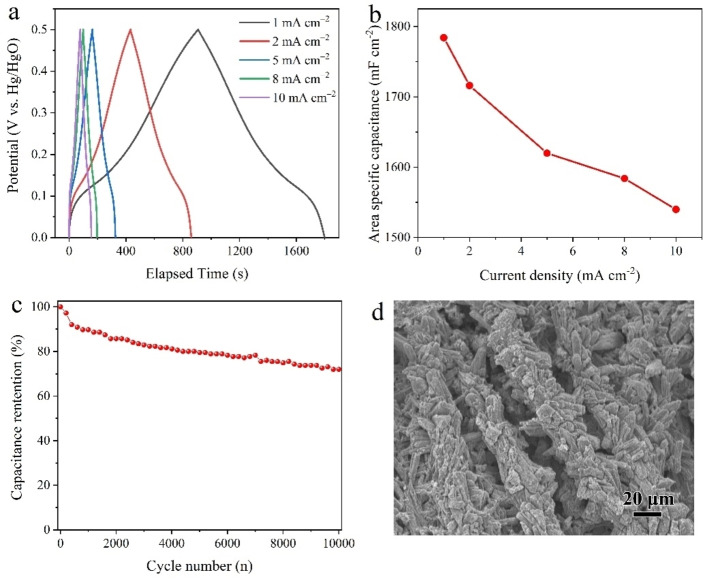
(a) Galvanostatic charge‐discharge curves and (b) corresponding areal specific capacitance of the Co‐MOF/CFC electrode at different current densities. (c) Cycling performance of the Co‐MOF/CFC electrode at a current density of 5 mA cm^−2^. (d) SEM image of the Co‐MOF/CFC electrode after cycling.

The GCD curves of Co‐MOF/CFC electrode within a potential window of 0–0.5 V at different current densities are shown in Figure 5a. The existence of voltage plateau in the charge‐discharge curves was in accordance with the redox peaks of CV curves (Figure 4a), further revealing the pseudocapacitive behavior of Co‐MOF/CFC. The areal and gravimetric specific capacitances of the Co‐MOF/CFC electrode were calculated from the discharge time based on Equations (5) and 6:

(5) $$C{}_{s}=I\Delta t/\left(S\Delta V\right)$$

(6) $$C{}_{m}=\left(I\Delta t\right)/\left(m\Delta V\right)$$

where *C*_s_ is the areal capacitance (F cm^−2^), *C*_m_ is the gravimetric capacitance (F g^−1^), *I* is the discharge current (A), Δ*t* is the discharge time (s), Δ*V* is the potential window (V), *m* is the mass (g) of active material, and *S* is the projected area of electrode (cm^2^). The Co‐MOF/CFC electrode delivered high areal specific capacitances of 1784, 1716, 1620, 1584 and 1540 mF cm^−2^ at current densities of 1, 2, 5, 8, and 10 mA cm^−2^, respectively (Figure 5b). The corresponding gravimetric specific capacitance were 137, 132, 125, 122 and 118 F g^−1^. With the increasing of current densities, the capacitances declined due to the increased internal diffusion resistance and relatively insufficient utilization of electroactive material at high current densities.^[41]^ Importantly, a high capacitance retention rate of 86 % was retained with a 10‐fold current increase from 1 to 10 mA cm^−2^, indicative of a superior rate capability. Furthermore, the electrochemical stability during the cycling was evaluated at 5 mA cm^−2^ (Figure 5c), over 70 % of the initial capacitance was remained after 10000 cycles. This good cycling stability was likely related to the excellent structural stability of Co‐MOF/CFC. The surface morphology of Co‐MOF/CFC electrode after the cycling was investigated by SEM (Figure 5d), which verifies that the structure of Co‐MOF/CFC was well maintained without significant deterioration or structure changes during charging/discharging processes. Overall, the areal/gravimetric specific capacitance and capacitance retention rate of Co‐MOF/CFC are comparable to or even superior to those of most reported MOF materials.[^9^, ^42^, ^43^, ^44^, ^45^, ^46^, ^47^, ^48^]

**Figure 6:**
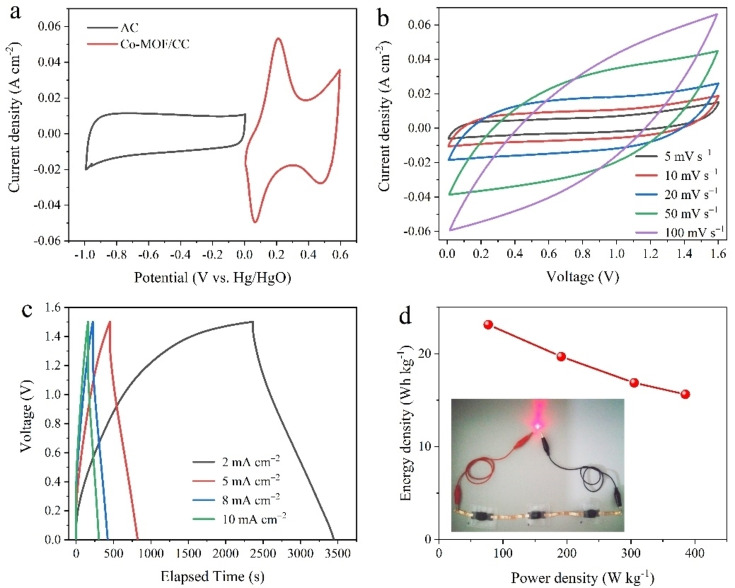
(a) CV curves for Co‐MOF/CFC and AC collected in different potential windows at a scan rate of 10 mV s^−1^. (b) CV curves of the Co‐MOF/CFC//AC ASSC device at various scan rates from 10 to100 mV s^−1^ over the 1.6 V potential window. (c) Galvanostatic charge‐discharge curves of the ASSC device at different current densities. (d) Ragone plot of the Co‐MOF/CFC//AC ASSC device. The inset is a digital photograph of a red LED powered by three ASSC devices connected in series.

To further evaluate the practical application of Co‐MOF/CFC, an asymmetric all‐solid‐state supercapacitor device (ASSC) was assembled with active carbon (AC) anode and PVA/LiOH (PVA, polyvinyl alcohol) gel electrolyte. The electrochemical performance of AC electrode was first tested using a three‐electrode configuration in 1 m LiOH. The rectangle‐like CV and triangle GCD curves of AC electrode confirmed the double‐layer capacitive behavior (Figure S7). The specific capacitance of AC was about 160 F g^−1^ at 5 mA cm^−2^. According to the charge balance between two electrodes, the loading mass ratio of Co‐MOF/CFC and AC was about 2. Figure 6a shows the CV curves of Co‐MOF/CFC and AC at 10 mV s^−1^, verifying a potential window of 0.6 V for Co‐MOF/CFC and 1.0 V for AC. So, it is expected that the working potential window of the assembled Co‐MOF/CFC//AC ASSC device can reach up to 1.6 V, which was verified (Figure S8). The CV curves of the ASSC at various scan rates (Figure 6b) were approximately rectangular. At low scan rate, broad oxidation and reduction peaks were observed, verifying the capacitance contribution from both electric double layer and pseudo capacitance.

The GCD curves of the ASSC at various current densities (Figure 6c) were in symmetric triangular shape even when the current density was increased to 10 mA cm^−2^, suggesting a good Coulombic efficiency and electrochemical reversibility. The specific capacitances based on the total mass of active materials on two electrodes were 74, 63, 54 and 50 F g^−1^ at 2, 5, 8 and 10 mA cm^−2^, respectively. A high capacitance retention rate of 68 % suggests a good rate capability. As shown in Figure S9, the ASSC retained more than 85 % of its initial specific capacitance after 1500 cycles at 10 mA cm^−2^. Additionally, the energy density (E, Wh kg^−1^) and power density (P, W kg^−1^) of the ASSC were calculated from GCD curves based on Equations (7) and 8:

(7) $$E=0.5C{}_{m}\left(\Delta V\right){}^{2}/3.6$$

(8) P=3600×E/Δt

where *C*_m_ is the gravimetric specific capacitance (F g^−1^), Δ*V* is the voltage window (V), and Δ*t* is the discharging time (s). As shown in the Ragone plot (Figure 6d), the ASSC exhibited a maximum energy density of 23.1 Wh kg^−1^ at a power density of 77.1 W kg^−1^, and still retained 15.6 Wh kg^−1^ at a maximum power density of 385.3 W kg^−1^. The maximum energy value was comparable to or larger than those of recently reported MOFs//AC SC devices, such as PNC//AC (16.0 Wh kg^−1^ at 749 W kg^−1^),^[49]^ 2D‐CMO//AC (36.4 Wh kg^−1^ at 90 W kg^−1^),^[15]^ Ni‐MOF/NF//AC (26.2 Wh kg^−1^ at 152.7 W kg^−1^),^[46]^ Ni/Co‐MOF//AC (12.8 Wh kg^−1^ at 372.5 W kg^−1^),^[50]^ and NiCo‐MOF NSHS//AC (20.94 Wh kg^−1^ at 750.84 W kg^−1^).^[51]^ Three ASSC devices connected in series could light up a red light emitting diode (LED) for 10 min (inset in Figure 6d). These results indicated that the Co‐MOF/CFC synthesized in this work could be well fitted in energy‐related fields.

## Conclusion

In summary, a facile and effective cathodic electrodeposition method has been developed for producing continuous uniform Co‐MOF coating on a CFC substrate, wherein 3D rodlike crystal bundles are coated onto the carbon fibers. The pre‐electrodeposited cobalt modifier has played a crucial role to provide active sites for nucleation and growth of MOF coating. The use of the electrodeposited highly‐active metal to promote the nucleation and growth of MOF is a new approach and is expected to be easily expanded to electrodeposit other MOF coatings on different conductive substrates. The obtained Co‐MOF/CFC electrodes have shown high areal capacitances, good cycling stability and improved rate capability, which can be attributed to the high mass loading, excellent structural stability and tight integration between MOF and CFC substrate. Such characteristics endow the CFC supported Co‐MOF coating with great potential toward applications such as energy storage, separation and catalysis.

## Experimental Section

### Material synthesis and characterization

The chemicals were purchased from Shanghai Aladdin Biochemical Technology Co., Ltd. and used without any further purification. CFC was purchased from CeTech Co., Ltd. and was sequentially pretreated by sonication in acetone, water and ethanol each for 20 min, followed by the activization in H_2_SO_4_/HNO_3_ (3 : 1 v/v) mixture at 70 °C for 3 h.

To electrodeposit Co‐MOF on CFC, a cobalt coating layer was pre‐electroplated at a current density of −10 mA cm^−2^ for 5 min from the CoSO_4_ aqueous solution (0.1 m) containing boric acid (0.2 m) and Na_2_SO_4_ (0.2 m) at room temperature. The solution pH value was 2. The obtained cobalt‐coated/modified electrodes (Co/CFC) were washed with water and ethanol, and then immediately immersed into the electrodeposition solution for growing Co‐MOF coating at a cathodic potential of −2.5 V for a designated timeframe. The electrolyte contained 5 mmol Co(NO_3_)_2_ ⋅ 6H_2_O, 2.5 mmol H_3_BTC and 1 mmol supporting electrolyte, triethylamine hydrochloride (Et_3_NHCl) in a mixed solvent of H_2_O (15 mL) and ethanol (15 mL). The final sample was denoted as Co‐MOF/CFC. The electrodeposition process were carried out in a two‐electrode cell with CFC or Co/CFC electrode as working electrode (cathode) and platinum plate as counter electrode (anode).

Samples were characterized using X‐ray diffraction (XRD, Bruker D8 Advance with Cu_Kα_ radiation), scanning electron microscopy (SEM, Hitachi SU8010), Fourier transform infrared spectroscopy (FTIR, Nicolet 460 spectrometer in the range of 4000–400 cm^−1^ using KBr pellets) and X‐ray photoelectron spectroscopy (XPS, Kratos Axis Ultra DLD with an Al_Kα_ X‐ray source). Thermogravimetric analysis (TGA) was carried out on a METTLER TGA‐DSC 1 apparatus at a heating rate of 10 °C min^−1^ under air.

### Electrochemical measurements

Electrochemical measurements were carried out in a three‐electrode system with Co‐MOF/CFC, Pt plate (1×1 cm^2^), Hg/HgO electrode and 1 m LiOH solution as the working electrode, counter electrode, reference electrode and electrolyte, respectively. Cyclic voltammetry (CV) was performed with a CHI 660D electrochemical workstation. The galvanostatic charge‐discharge tests (GCD) were conducted using a LAND CT2001A instrument.

### Fabrication of supercapacitor devices

The supercapacitor device was fabricated with Co‐MOF/CFC (1×1 cm^2^) as positive electrode, activated carbon (AC) as negative electrode, and polymer gel (PVA/LiOHl) as electrolyte and separator. Negative electrodes were prepared by mixing AC, acetylene black and polyvinylidene fluoride (PVDF) at a mass ratio of 8 : 1 : 1 in N‐methyl pyrrolidone (NMP). The resulting slurry was pasted on CFC (1×1 cm^2^) and dried at 70 °C for 12 h. The mass loading of AC was determined by balancing the charges between positive and negative electrodes (*Q*_+_=*Q*_−_) according to Equation 9:

(9) $$m{}_{+}/m{}_{-}=\left(\Delta V\times C{}_{-}\right)/\left(\Delta V\times C{}_{+}\right)$$

where *C* and *m* stand for the specific capacitance and active material mass of the positive (+) and negative (−) electrodes, respectively; Δ*V* is the potential window. The PVA/LiOH gel electrolyte was prepared as follows: PVA powder (3 g) was dissolved in H_2_O (12 mL) under vigorous stirring at 85 °C until the mixture became a clear and transparent solution. After cooling down to 60 °C, LiOH aqueous solution (4 m, 8 mL) was slowly added to the solution and stirred evenly.

## Conflict of interest

The authors declare no conflict of interest.

1

## Supporting information

As a service to our authors and readers, this journal provides supporting information supplied by the authors. Such materials are peer reviewed and may be re‐organized for online delivery, but are not copy‐edited or typeset. Technical support issues arising from supporting information (other than missing files) should be addressed to the authors.

Supporting Information

## Data Availability

The data that support the findings of this study are available from the corresponding author upon reasonable request.

## References

[1] W. Li , X. Zhao , Q. Bi , Q. Ma , L. Han , K. Tao , Dalton Trans. 2021, 50, 11701. 34382980 10.1039/d1dt02066h

[2] N. Raza , T. Kumar , V. Singh , K.-H. Kim , Coord. Chem. Rev. 2021, 430, 213660.

[3] S. Zheng , Q. Li , H. Xue , H. Pang , Q. Xu , Natl. Sci. Rev. 2020, 7, 305–314. 34692046 10.1093/nsr/nwz137 PMC8288962

[4] Y. Li , Y. Shan , H. Pang , Chin. Chem. Lett. 2020, 31, 2280–2286.

[5] C. Liu , Y. Bai , W. Li , F. Yang , G. Zhang , H. Pang , Angew. Chem. Int. Ed. 2022, 61, e202116282. 10.1002/anie.202116282 35005827

[6] L. Zhong , J. Ding , J. Qian , M. Hong , Coord. Chem. Rev. 2021, 434, 213804.

[7] J. Meng , X. Liu , C. Niu , Q. Pang , J. Li , F. Liu , Z. Liu , L. Mai , Chem. Soc. Rev. 2020, 49, 3142–3186. 32249862 10.1039/c9cs00806c

[8] S. D. Worrall , H. Mann , A. Rogers , M. A. Bissett , M. P. Attfield , R. A. W. Dryfe , Electrochim. Acta 2016, 197, 228–240.

[9] L. Wang , X. Feng , L. Ren , Q. Piao , J. Zhong , Y. Wang , H. Li , Y. Chen , B. Wang , J. Am. Chem. Soc. 2015, 137, 4920–4923. 25864960 10.1021/jacs.5b01613

[10] H. Al-Kutubi , J. Gascon , E. J. R. Sudhölter , L. Rassaei , ChemElectroChem 2015, 2, 462–474.

[11] X. Shi , Y. Shan , M. Du , H. Pang , Coord. Chem. Rev. 2021, 444, 214060.

[12] T. R. C. Van Assche , G. Desmet , R. Ameloot , D. E. De Vos , H. Terryn , J. F. M. Denayer , Microporous Mesoporous Mater. 2012, 158, 209–213.

[13] A. Martinez Joaristi , J. Juan-Alcañiz , P. Serra-Crespo , F. Kapteijn , J. Gascon , Cryst. Growth Des. 2012, 12, 3489–3498.

[14] N. Campagnol , T. R. C. Van Assche , M. Li , L. Stappers , M. Dincă , J. F. M. Denayer , K. Binnemans , D. E. De Vos , J. Fransaer , J. Mater. Chem. A 2016, 4, 3914–3925.

[15] X. Zhang , J. Luo , P. Tang , X. Ye , X. Peng , H. Tang , S.-G. Sun , J. Fransaer , Nano Energy 2017, 31, 311–321.

[16] M. Li , M. Dinca , J. Am. Chem. Soc. 2011, 133, 12926–12929. 21790152 10.1021/ja2041546

[17] L. Wang , Y. Wu , R. Cao , L. Ren , M. Chen , X. Feng , J. Zhou , B. Wang , ACS Appl. Mater. Interf. 2016, 8, 16736–16743. 10.1021/acsami.6b05375 27300143

[18] S. Alizadeh , D. Nematollahi , J. Am. Chem. Soc. 2017, 139, 4753–4761. 28291345 10.1021/jacs.6b12564

[19] Q. Liu , N. Wang , J. Caro , A. Huang , J. Am. Chem. Soc. 2013, 135, 17679–17682. 24224527 10.1021/ja4080562

[20] S. Qiu , M. Xue , G. Zhu , Chem. Soc. Rev. 2014, 43, 6116–6140. 24967810 10.1039/c4cs00159a

[21] Y. Liu , N. Wang , J. H. Pan , F. Steinbach , J. Caro , J. Am. Chem. Soc. 2014, 136, 14353–14356. 25280264 10.1021/ja507408s

[22] Y. Liu , J. H. Pan , N. Wang , F. Steinbach , X. Liu , J. Caro , Angew. Chem. Int. Ed. 2015, 54, 3028—3032; 10.1002/anie.201411550 25611948

[23] X. Zhang , Y. Liu , L. Kong , H. Liu , J. Qiu , W. Han , L.-T. Weng , K. L. Yeung , W. Zhu , J. Mater. Chem. A 2013, 1, 10635–10638.

[24] S. Li , C. Yu , J. Yang , C. Zhao , M. Zhang , H. Huang , Z. Liu , W. Guo , J. Qiu , Energy Environ. Sci. 2017, 10, 1958–1965.

[25] S. Zhou , Y. Wei , L. Zhuang , L.-X. Ding , H. Wang , J. Mater. Chem. A 2017, 5, 1948–1951.

[26] O. M. Yaghi , H. Li , T. L. Groy , J. Am. Chem. Soc. 1996, 118, 9096–9101. https://www.ccdc.cam.ac.uk/services/structures?id=doi:10.1021/ja960746q contains the supplementary crystallographic data for this paper. These data are provided free of charge by the joint Cambridge Crystallographic Data Centre and Fachinformationszentrum Karlsruhe http://www.ccdc.cam.ac.uk/structures Access Structures service.

[27] S. Shamaei , A. R. Abbasi , N. Noori , E. Rafiee , A. Azadbakht , Colloids Surf. A 2013, 431, 66–72.

[28] X. Hu , H. Hu , C. Li , T. Li , X. Lou , Q. Chen , B. Hu , J. Solid State Chem. 2016, 242, 71–76.

[29] C. Li , X. Lou , M. Shen , X. Hu , Z. Guo , Y. Wang , B. Hu , Q. Chen , ACS Appl. Mater. Interfaces 2016, 8, 15352–15360. 27142789 10.1021/acsami.6b03648

[30] X. Zhang , N. Qu , S. Yang , Q. Fan , D. Lei , A. Liu , X. Chen , J. Colloid Interface Sci. 2020, 575, 347–355. 32388026 10.1016/j.jcis.2020.04.127

[31] J. Linnemann , L. Taudien , M. Klose , L. Giebeler , J. Mater. Chem. A 2017, 5, 18420–18428.

[32] D. O. Miles , D. Jiang , A. D. Burrows , J. E. Halls , F. Marken , Electrochem. Commun. 2013, 27, 9–13.

[33] C. Qu , Y. Jiao , B. Zhao , D. Chen , R. Zou , K. S. Walton , M. Liu , Nano Energy 2016, 26, 66–73.

[34] S. H. Kazemi , B. Hosseinzadeh , H. Kazemi , M. A. Kiani , S. Hajati , ACS Appl. Mater. Interfaces 2018, 10, 23063–23073. 29882650 10.1021/acsami.8b04502

[35] F. Ghamari , D. Raoufi , S. Alizadeh , J. Arjomandi , D. Nematollahi , J. Mater. Chem. A 2021, 9, 15381–15393.

[36] J. Liu , J. Wang , C. Xu , H. Jiang , C. Li , L. Zhang , J. Lin , Z. X. Shen , Adv. Sci. 2018, 5, 1700322. 10.1002/advs.201700322 PMC5770679 29375964

[37] F. Wang , X. Wu , X. Yuan , Z. Liu , Y. Zhang , L. Fu , Y. Zhu , Q. Zhou , Y. Wu , W. Huang , Chem. Soc. Rev. 2017, 46, 6816–6854. 28868557 10.1039/c7cs00205j

[38] H. Liu , H. Guo , W. Yao , L. Zhang , M. Wang , T. Fan , W. Yang , W. Yang , Colloids Surf. A 2020, 601, 125011.

[39] P.-P. Sun , Y.-H. Zhang , X. Yu , Q. Shi , B. Tian , J. Gao , F.-N. Shi , Inorg. Chim. Acta 2020, 508, 119629.

[40] Q. Li , H. Guo , R. Xue , M. Wang , M. Xu , W. Yang , J. Zhang , W. Yang , Int. J. Hydrogen Energy 2020, 45, 20820–20831.

[41] F. Meng , Z. Fang , Z. Li , W. Xu , M. Wang , Y. Liu , J. Zhang , W. Wang , D. Zhao , X. Guo , J. Mater. Chem. A 2013, 1, 7235.

[42] X. Du , J. Zhang , H. Wang , Z. Huang , A. Guo , L. Zhao , Y. Niu , X. Li , B. Wu , Y. Liu , Mater. Chem. Front. 2020, 4, 243–251.

[43] K. Wang , B. Lv , Z. Wang , H. Wu , J. Xu , Q. Zhang , Dalton Trans. 2020, 49, 411–417. 31833493 10.1039/c9dt04101j

[44] R. Hou , M. Miao , Q. Wang , T. Yue , H. Liu , H. S. Park , K. Qi , B. Y. Xia , Adv. Energy Mater. 2020, 10, 1901892.

[45] Z. Li , Y. Sun , R. Hu , S. Ye , J. Song , L. Liu , J. Qu , RSC Adv. 2021, 11, 8362–8366. 35423289 10.1039/d1ra00259g PMC8695202

[46] D. Zheng , H. Wen , X. Sun , X. Guan , J. Zhang , W. Tian , H. Feng , H. Wang , Y. Yao , Chem. Eur. J. 2020, 26, 17149–17155. 32767604 10.1002/chem.202003220

[47] Y.-F. Wang , S.-Y. Yang , Y. Yue , S.-W. Bian , J. Alloys Compd. 2020, 835, 155238.

[48] D. Sheberla , J. C. Bachman , J. S. Elias , C. J. Sun , Y. Shao-Horn , M. Dinca , Nat. Mater. 2017, 16, 220–224. 27723738 10.1038/nmat4766

[49] K. Wang , R. Bi , M. Huang , B. Lv , H. Wang , C. Li , H. Wu , Q. Zhang , Inorg. Chem. 2020, 59, 6808–6814. 32330019 10.1021/acs.inorgchem.0c00060

[50] S. Sun , M. Huang , P. Wang , M. Lu , J. Electrochem. Soc. 2019, 116, A1799-A1805.

[51] J. Sun , X. Yu , S. Zhao , H. Chen , K. Tao , L. Han , Inorg. Chem. 2020, 59, 11385–11395. 32799472 10.1021/acs.inorgchem.0c01157

