# Supplementary material for: Efficient Metal‐Oriented Electrodeposition of a Co‐Based Metal‐Organic Framework with Superior Capacitive Performance
Source: ChemSusChem. 2022 May 24;15(14):e202200644. doi: 10.1002/cssc.202200644 (PMC9401579; doi:10.1002/cssc.202200644)
Supplement: Supplementary file 1 — Supporting Information [file CSSC-15-0-s001.pdf]

ChemSusChem

Supporting Information

Efficient Metal-Oriented Electrodeposition of a Co-Based Metal-Organic Framework with Superior Capacitive Performance

Yan Han, Jian Cui, Yue Yu, Yunfeng Chao, Dejun Li,* Caiyun Wang,* and Gordon G. Wallace© 2022 The Authors. ChemSusChem published by Wiley-VCH GmbH. This is an open access article under the terms of the Creative Commons Attribution License, which permits use, distribution and reproduction in any medium, provided the original work is properly cited.

Figure S1. Digital photographs of electrodeposited Co-MOF on CFC. (a) Co-MOF on unmodified CFC deposited for 15 min; (b) Co-MOF on unmodified CFC deposited for 30 min; (c) Co-MOF on Co-modified CFC deposited for 15 min.

Figure S2. XRD pattern of Co/CFC.

Figure S3. SEM images of blank CFC (a, b) and Co/CFC (c, d).

Figure S4. SEM images of Co-MOF/CFC electrodeposited for 1 min (a, b), 5 min (c, d), 10 min (e, f), 20 min (g) and 30 min (h).

Figure S5. High-solution C1s XPS spectrum of Co-MOF/CC.

Figure S6. CV curves of Co-MOF/CFC and Co/CFC electrodes at 20 mV s^{-1}.

Figure S7. (a) CV curves of AC electrode at different scan rates. (b) Galvanostatic charge-discharge curves of AC electrode at different current densities.

Figure S8. CV curves of Co-MOF/CFC//AC ASSC device collected in different potential windows at a scan rate of 10 mV s^{-1}.

Figure S9. Cycling performance of Co-MOF/CFC//AC ASSC device at a current density of 10 mA cm^{-2}.

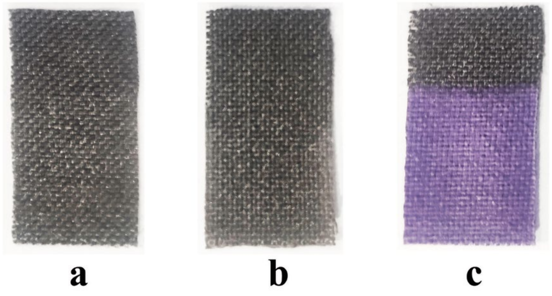

Figure S1. Digital photographs of electrodeposited Co-MOF on bare CFC and Co-modied CFC: (a) on unmodified CFC deposited for 15 min; (b) on unmodified CFC deposited for 30 min; (c) on Co-modified CFC deposited for 15 min.

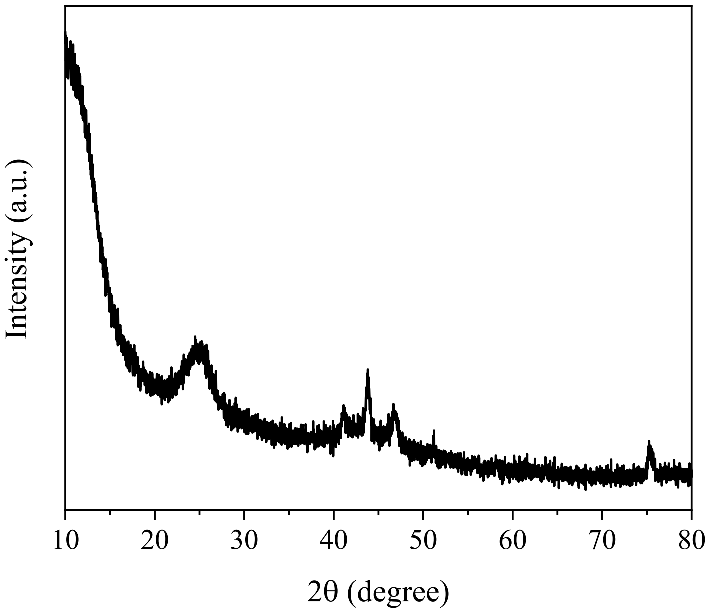

Figure S2. XRD pattern of Co/CFC.

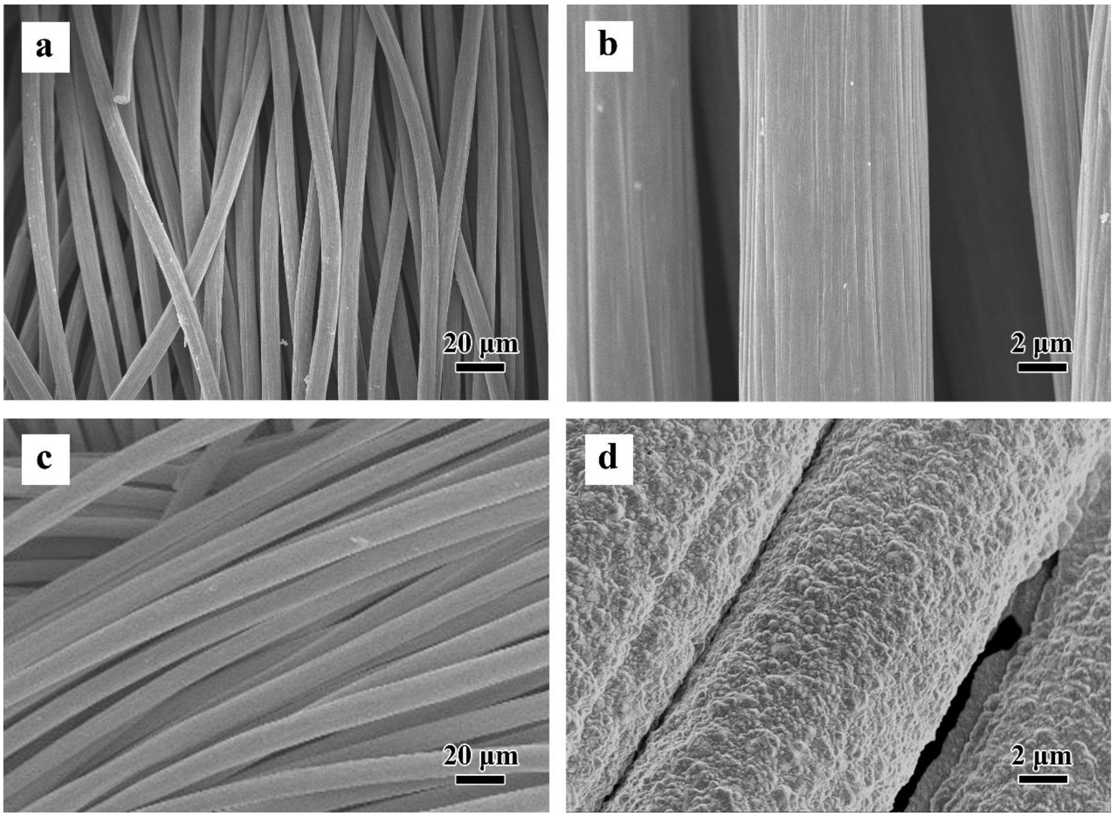

Figure S3. SEM images of blank CFC (a, b) and Co/CFC (c, d).

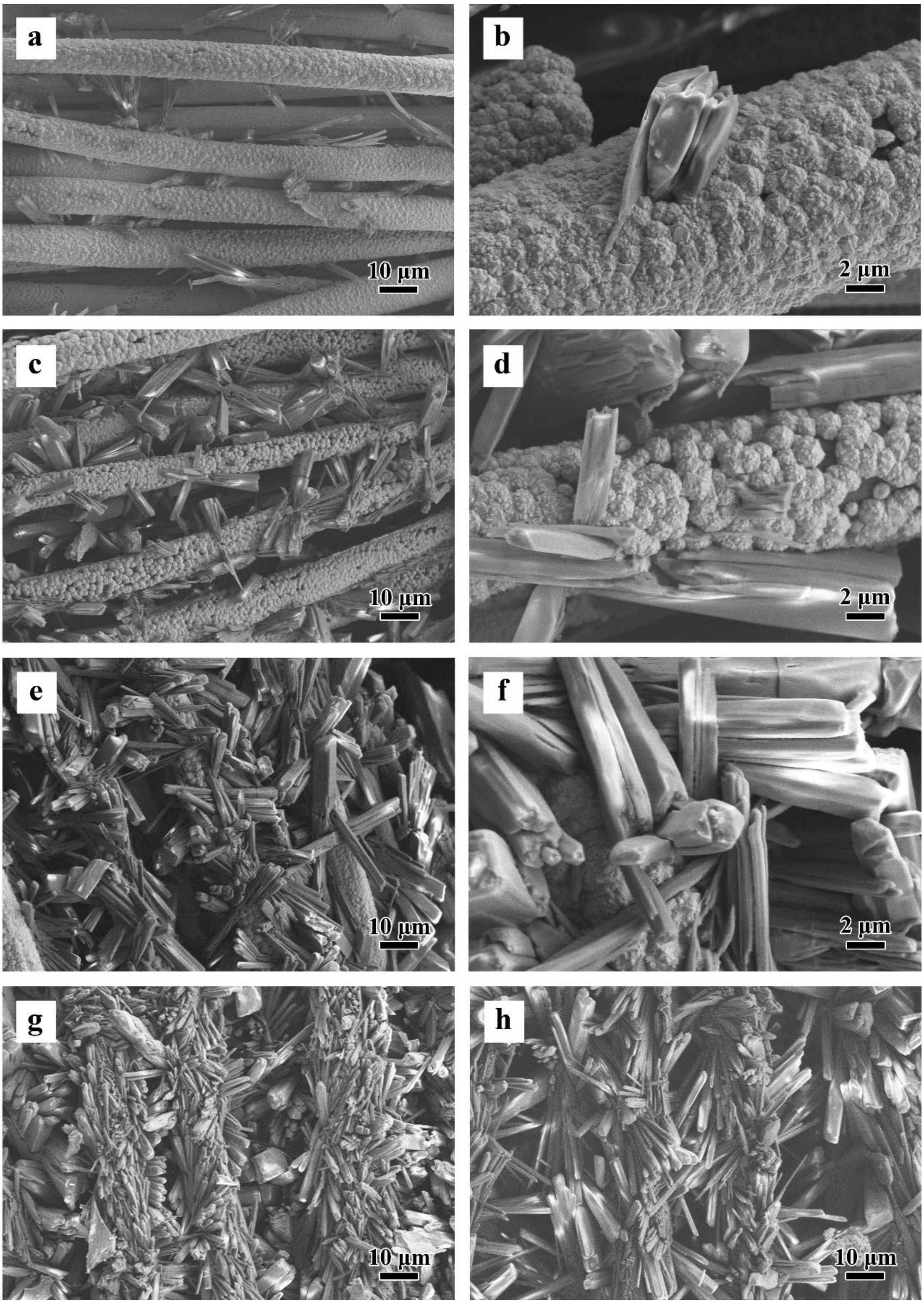

Figure S4. SEM images of Co-MOF/CFC electrodeposited for 1 min (a, b), 5 min (c, d), 10 min (e, f), 20 min (g) and 30 min (h).

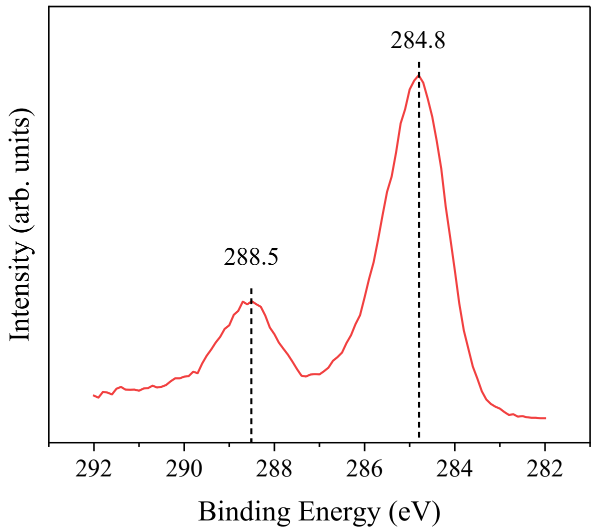

Figure S5. High-solution C1s XPS spectrum of Co-MOF/CC.

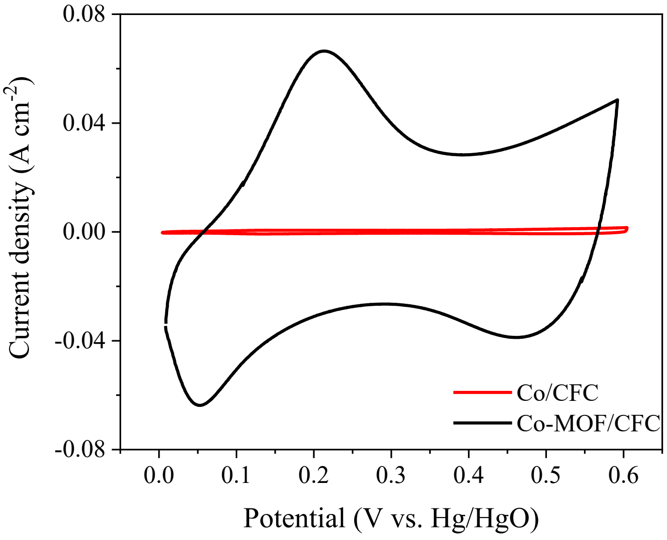

Figure S6. CV curves of Co-MOF/CFC and Co/CFC electrodes at 20 mV s^{-1}.

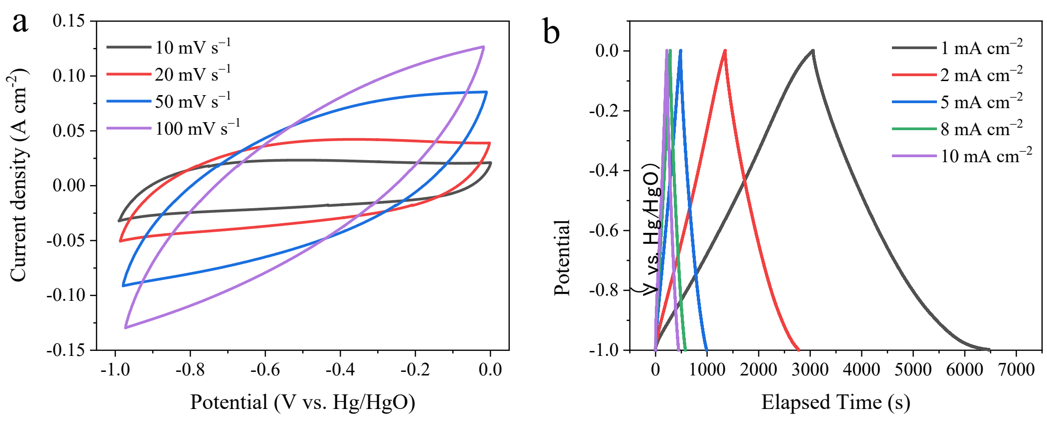

Figure S7. (a) CV curves of AC electrodes at different scan rates. (b) Galvanostatic charge-discharge curves of AC electrodes at different current densities.

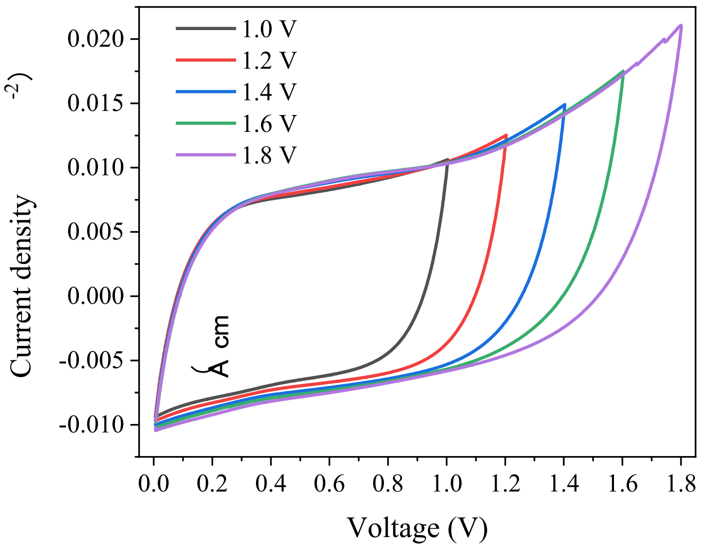

Figure S8. CV curves of Co-MOF/CFC//AC ASSC device collected over different potential windows at a scan rate of 10 mV s^{-1}.

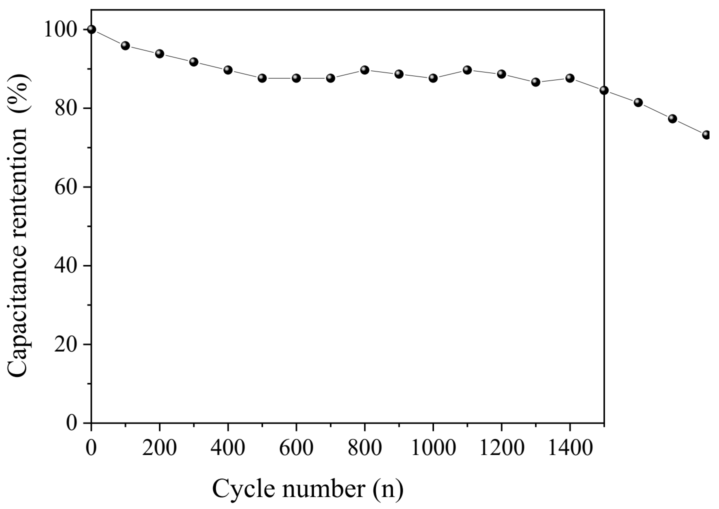

Figure S9. Cycling performance of Co-MOF/CFC//AC ASSC device at a current density of 10 mA cm^{-2}.
